# Hypoxia alters expression of Zebrafish Microtubule-associated protein Tau (*mapta, maptb*) gene transcripts

**DOI:** 10.1186/1756-0500-7-767

**Published:** 2014-10-31

**Authors:** Seyyed Hani Moussavi Nik, Morgan Newman, Swamynathan Ganesan, Mengqi Chen, Ralph Martins, Giuseppe Verdile, Michael Lardelli

**Affiliations:** Discipline of Genetics, School of Molecular and Biomedical Sciences, The University of Adelaide, SA 5005 Adelaide, Australia; Centre of Excellence for Alzheimer’s disease Research and Care, School of Medical Sciences, Edith Cowan University, Joondalup, WA Australia; School of Psychiatry and Clinical Neurosciences, University of Western Australia, Crawley, WA Australia; McCusker Alzheimer’s Disease Research Foundation, Hollywood Private Hospital, Perth, WA Australia; School of Biomedical Sciences, Faculty of Health Sciences, Curtin University, Bentley, WA Australia; Zebrafish Genetics Laboratory, School of Molecular and, Biomedical Sciences, The University of Adelaide, Adelaide, SA 5005 Australia

**Keywords:** Microtubule-associated protein tau *(MAPT*), Alternative splicing, Alzheimer’s disease, Hypoxia, Zebrafish

## Abstract

**Background:**

Microtubule-associated protein tau (*MAPT*) is abundant in neurons and functions in assembly and stabilization of microtubules to maintain cytoskeletal structure. Human *MAPT* transcripts undergo alternative splicing to produce 3R and 4R isoforms normally present at approximately equal levels in the adult brain. Imbalance of the 3R-4R isoform ratio can affect microtubule binding and assembly and may promote tau hyperphosphorylation and neurofibrillary tangle formation as seen in neurodegenerative diseases such as frontotemporal dementia (FTD) and Alzheimer’s disease (AD). Conditions involving hypoxia such as cerebral ischemia and stroke can promote similar tau pathology but whether hypoxic conditions cause changes in MAPT isoform formation has not been widely explored. We previously identified two paralogues (co-orthologues) of *MAPT* in zebrafish, *mapta* and *maptb*.

**Results:**

In this study we assess the splicing of transcripts of these genes in adult zebrafish brain under hypoxic conditions. We find hypoxia causes increases in particular *mapta* and *maptb* transcript isoforms, particularly the 6R and 4R isoforms of *mapta* and *maptb* respectively. Expression of the zebrafish orthologue of human *TRA2B*, *tra2b*, that encodes a protein binding to MAPT transcripts and regulating splicing, was reduced under hypoxic conditions, similar to observations in AD brain.

**Conclusion:**

Overall, our findings indicate that hypoxia can alter splicing of zebrafish *MAPT* co-orthologues promoting formation of longer transcripts and possibly generating Mapt proteins more prone to hyperphosphorylation. This supports the use of zebrafish to provide insight into the mechanisms regulating *MAPT* transcript splicing under conditions that promote neuronal dysfunction and degeneration.

## Background

The *MICROTUBULE-ASSOCIATED PROTEIN TAU* (*MAPT*) gene encodes the soluble tau protein that is abundant in neurons and functions to assemble and stabilize microtubules to maintain cytoskeletal structure [1]. As a result of alternative splicing of *MAPT* transcripts, six tau protein isoforms ranging from 352 to 441 amino acid residues in length are generated and expressed in the human brain. The isoforms differ by the regulated inclusion or exclusion of two regions of sequence near the N-terminus and the possession of either three (3R) or four (4R) repeat regions, (corresponding to the microtubule-binding domains), towards the C-terminus of tau [2]. The 3R isoform is generated from mRNAs lacking exon 10, while mRNAs containing exon 10 encode 4R tau. These isoforms are normally present at approximately equal levels in the adult human brain [3]. Changes in this isoform ratio and post-translational modifications of the 3R and 4R isoforms affect microtubule binding and assembly [4, 5].

Dysregulation of tau splicing is often observed in neurodegenerative diseases with aberrant tau deposition, including frontotemporal dementia (FTD), Pick disease (PiD), progressive supranuclear palsy (PSP) [6] and Alzheimer’s disease (AD) [7]. Mutations reported in FTD cause aberrant exon 10 splicing, resulting in altered 4R/3R tau ratios [8, 9]. In PSP, aggregates of 4R tau predominate, whereas 3R isoforms are found in excess in Pick bodies in the majority of cases of PiD [10, 11]. In AD brains, increases in 4R tau isoforms have been reported resulting in altered 4R/3R tau ratios [12]. Neurofibrillary tangles (NFTs), a major pathological hallmark of the AD brain, can result from the phosphorylation of 3R tau, 4R tau or both [13, 14]. Thus, any alternations in the levels of these isoforms could promote tangle formation and disease progression. It should be noted that changes in tau protein isoform ratios could result both from changes in the alternative splicing of transcripts and differential changes in the stability of their protein products.

Conditions such as cerebral ischemia and stroke that result in hypoxic conditions in affected brain areas can promote tau hyperphosphorylation and formation of NFTs. Acute hypoxic conditions have been shown to activate kinases that phosphorylate tau resulting in accumulation of phosphorylated tau in neurons [15]. In a rodent stroke model, hyperphosphorylated tau accumulated in neurons of the cerebral cortex in areas where ischemic damage was prominent. This was associated with the up-regulation of the tau phosphorylating enzyme CdK5, and the consequent promotion of the formation of filaments similar to those present in human neurodegenerative tauopathies [16]. It stands to reason that increases in tau isoforms may also contribute to this process by increasing the availability of the tau substrate to phosphorylating enzymes.

The zebrafish, *Danio rerio*, is an emerging model organism for the study of neurodegenerative disease [17]. Zebrafish embryos represent normal collections of cells in which complex and subtle manipulations of gene activity can be performed to facilitate analyses of genes involved in human disease. The zebrafish genome is extensively annotated and regions of conservation of chromosomal synteny between humans and zebrafish have been defined [18]. In many cases zebrafish genes are identifiable that are clear orthologues of human genes. For example, the AD-relevant *PRESENILIN* genes (*PSEN1* and *PSEN2)* have zebrafish orthologues of *psen1* [19] and *psen2* [20] respectively. Tau phosphorylation and subsequent toxicity has been reported in zebrafish over-expressing the FTD associated human tau mutation, P301L [21, 22]. However this model does not reflect the pathology of other dementias such as AD where factors that regulate levels of wild-type tau isoforms promote hyper-phosphorylation and neurodegeneration.

**Figure 1:**
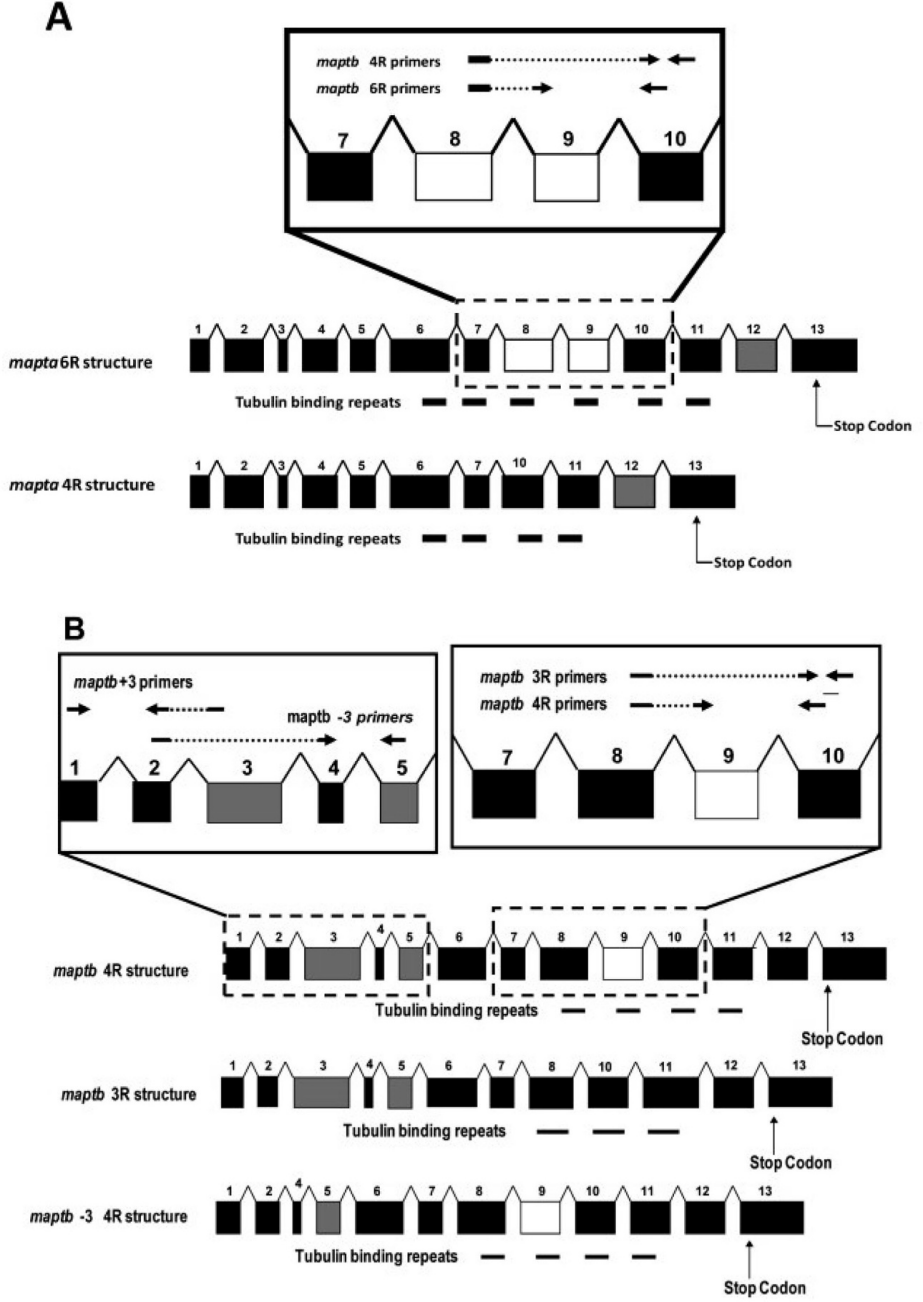
**Splicing isoforms of** ***mapta*** **and** ***maptb*** **mRNA transcripts.** Grey and white boxes indicate exons subject to alternative splicing. The black lines below exons indicate those encoding tubulin-binding motifs. Arrows indicate the approximate binding sites of primers used in qPCR analyses of splicing isoforms. **(A)** Exon structure of *mapta*.isoforms **(B)** Exon structure of *maptb* isoforms.

We have previously identified two paralogues (co-orthologues) of *MAPT* in zebrafish, denoted *mapta* and *maptb* and have shown that both genes are expressed in the developing central nervous system [23]. (Teleosts appear to have undergone an additional round of genome duplication since their separation from the tetrapod lineage followed by loss of many of the duplicated genes [18]). Similar to human *MAPT*, a complex pattern of alternative splicing of the *mapta* and *maptb* transcripts occurs. Zebrafish *mapta* gives rise to transcripts encoding 4R-6R isoforms, whereas *maptb* is predominantly expressed as a 3R isoform [23] (Figure 1) and is also alternatively spliced to form a “big tau” isoform. In mammals “big tau” is expressed in the peripheral nervous system and other tissues [24–26] while in zebrafish we observed “big tau” expression (at 24 hours post fertilization, hpf) in the trigeminal ganglion and dorsal spinal cord neurons (possibly dorsal sensory neurons) [23]. However, whether hypoxic conditions lead to changes in tau isoform expression has not been widely explored in zebrafish. In the work described in this paper we extend our examination of expression of the zebrafish tau co-orthologues to study their response to actual hypoxia in adult fish brains and to chemical mimicry of hypoxia in explanted adult fish brains. We observe increases in the overall levels of both *mapta* and *maptb* transcripts due to specific increases in the levels of *mapta* 6R and *maptb* 4R transcript isoforms. This is consistent with dramatically decreased levels of transcripts of the zebrafish orthologue of the human *TRA2B* gene that codes for a splicing factor regulating alternative splicing of *MAPT* transcripts in human cells [12]. We also observe an apparent increase under hypoxia in the levels of shorter transcripts of *maptb* relative to “big tau” transcripts of this gene. Overall, our findings indicate that hypoxia can alter splicing of zebrafish MAPT co-orthologues promoting formation of longer transcripts and possibly generating Mapt proteins more prone to hyperphosphorylation. This supports the use of zebrafish to provide insight into the mechanisms regulating *MAPT* transcript splicing under conditions that promote neuronal dysfunction and degeneration.

## Results

To determine whether hypoxic conditions regulate alternative splicing in *MAPT* co-orthologues in zebrafish, levels of *mapta* and *maptb* transcripts were assessed in adult zebrafish brains under conditions of actual hypoxia or in explanted adult brains subjected to chemical mimicry of hypoxia caused by NaN_3_.

**Figure 2:**
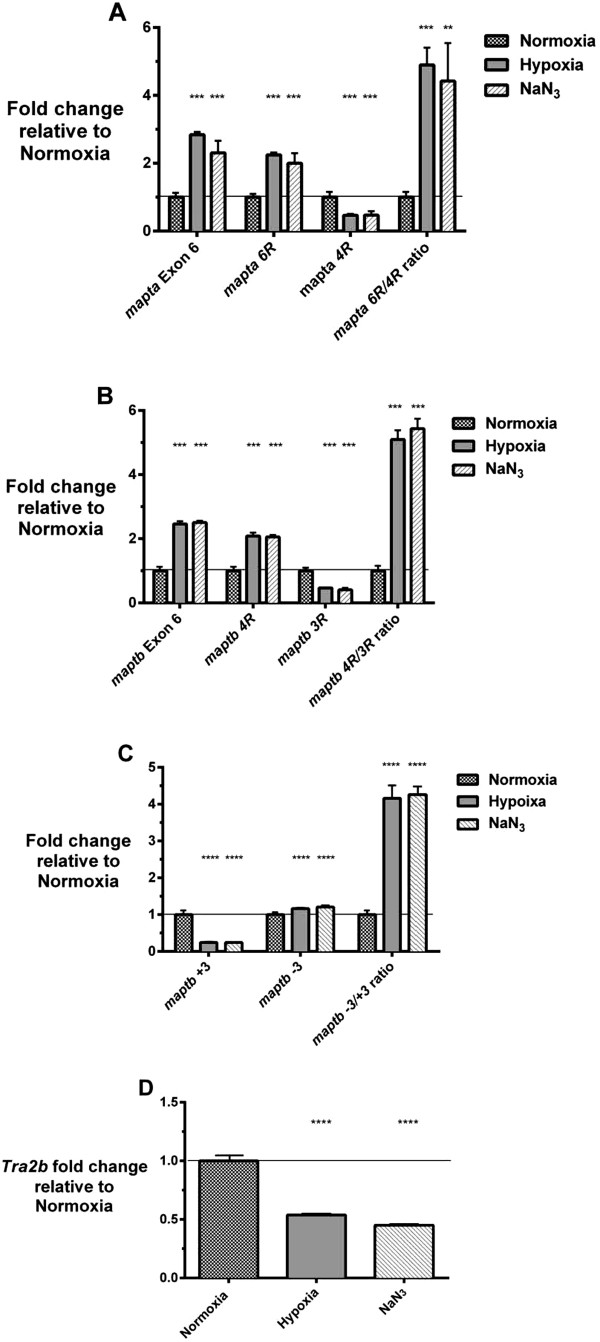
**qPCR analyses of the expression of A) Measurement of** ***mapta*** **exon 6 levels gives the combined expression of all** ***mapta*** **transcripts in zebrafish brains.** qPCRs to determine relative *mapta* 6R and 4R isoform levels show increased and decreased expression under hypoxia respectively. **B)** Measurement of *maptb* exon 6 levels gives the combined expression of all *maptb* transcripts in zebrafish brains*.* qPCRs to determine relative *maptb* 4R and 3R isoform levels show increased and decreased expression under hypoxia respectively. **C)** *maptb +3* (“big tau”) is decreased relative to *maptb −3* under hypoxia. **D)** *tra2b* transcript levels under normoxia are higher relative to those under hypoxia or chemical mimicry of hypoxia (sodium azide exposure). Expression ratios for *mapta and maptb* are shown relative to normoxia (the normoxia expression level is normalized to *eef1a1l1*). ***P ≤ 0.0001; **P ≤ 0.001; ****P ≤ 0.00001. Error bars represent standard error of the mean.

In studies of hypoxia it is common to use chemical agents that can mimic (partially) hypoxic conditions (also known as “chemical hypoxia”). Agents commonly used are cobalt chloride (CoCl_2_), nickel chloride (NiCl_2_) and NaN_3_. Azides, including NaN_3_, have an action on the respiratory chain very similar to that of cyanide. We have previously shown that exposure to aqueous solutions of NaN_3_ can induce hypoxia-like responses in zebrafish [27]. Exposure of adult fish to hypoxia or exposure of explanted adult brains to chemical mimicry of hypoxia increases the overall expression of tau transcripts in zebrafish brains. This was shown by qPCR measurement involving amplification of exonic sequence included in all transcripts of *mapta* or *maptb* (i.e. exon 6 of both genes – see Figure 2A and 2B). We also observed that the pattern of tau transcript splicing differs between hypoxia-exposed brains and controls. In terms of contributing isoforms, expression of the *mapta 6R* isoform was significantly increased, while expression of the *mapta 4R* isoform showed a significant decrease under hypoxia (Figure 2A). We also observed a significantly increased level of expression of *maptb 4R* transcripts, while expression of *maptb 3R* transcripts also showed a significant decrease under hypoxia (Figure 2B). An increase in expression of *maptb 4R* but not *3R* corresponds to an overall increase in the 4R/3R ratio of tau transcripts (Figure 2B).

In rats (and humans) *Mapt* exon 4a contains a large open reading frame. Inclusion of this exonic sequence in *MAPT* mRNAs allows translation of “big tau” protein. Exon 3 of zebrafish *maptb* appears to be equivalent to rat exon 4a in size although no sequence homology is observed. Like rat *MAPT* exon 4a, zebrafish *maptb* exon 3 is subject to alternative splicing [23]. Therefore, we performed qPCR to test whether this alternative splicing event is also influenced by hypoxic conditions. We observed that exclusion of exon 3 (here denoted as *maptb −*3) from zebrafish *maptb* transcripts is significantly increased under hypoxia and chemical mimicry of hypoxia when compared with inclusion of exon 3 (here denoted as *maptb +3*) (Figure 2C).

**Figure 3:**
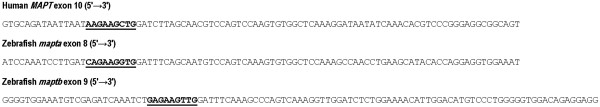
**Sequences from human** ***MAPT*** **and zebrafish** ***mapta*** **and** ***maptb*** **were analysed for the presence of possible Tra2B binding sites using the online software ESE finder (** http://genes.mit.edu/burgelab/rescue-ese/ **).** Bold, underlined letters are putative Tra2b-binding sites.

In humans, differential splicing of *MAPT* transcripts in response to hypoxia can occur due to decreased binding of TRA2 protein to RNA [28]. The *TRA2* gene is duplicated in vertebrates, resulting in two TRA2 proteins with aprpoximately 63% amino acid residue identity in humans [29]. These proteins are denoted TRA2A encoded by the *TRA2A* gene and TRA2B protein encoded by the gene *TRA2B* (also known as *SFRS10*). Nuclear magnetic resonance (NMR) analyses have recently shown that the optimal core RNA target sequence for binding TRA2B protein is AGAA. Conrad *et al*. [12] observed AD-specific changes in TRA2B expression, suggesting a potential mechanism for altered tau in AD. Suh *et al.* [28] also observed a decrease in mouse Tra2b expression leading to a decrease in exon 10 exclusion and 3R-tau expression in cortical neurons after transient occlusion of the middle cerebral artery in mice. To examine whether this behavior is conserved for the zebrafish *mapta* and *maptb* genes we first observed whether hypoxia alters expression of the *TRA2B* orthologous gene, *tra2b*, in zebrafish brains. As shown in Figure 2D both actual hypoxia and chemical mimicry of hypoxia lead to decreased *tra2b* transcript levels presumably indicating reduction in the splice-regulating activity of Tra2b protein. We then examined whether the zebrafish *mapta* and *maptb* genes possess potential Tra2b binding sites within exons encoding tubulin-binding repeats and subject to alternative splicing. Using the online software, ESE finder (http://genes.mit.edu/burgelab/rescue-ese/) [30], zebrafish sequences for *mapta* exon 8 and *maptb* exon 9 were examined for putative tra2b binding sites. We found multiple exonic splicing enhancers (ESEs) but, for each gene, only one appeared significantly similar to the human TRA2B-binding site (Figure 3).

## Discussion

The human *MAPT* gene is located on chromosome 17 and contains 16 exons. Alternative splicing of the primary transcript leads to a family of mRNAs, encoding different protein isoforms. In adult human brain, six isoforms are expressed, produced by alternative splicing of exons 2, 3, and 10. Tau isoforms in the CNS contain either three or four copies of a tandem repeat containing tubulin-binding sequences (encoded by exon 10), referred to as 3R and 4R-tau [24]. Optional inclusion of exon 2, or exons 2 and 3, gives rise to N-terminal inclusions of 29 or 58 amino acid residues respectively [24].

In this study we provide evidence that exposure to actual hypoxia and to chemical mimicry of hypoxia leads to overall increases in tau transcript levels and, simultaneously, marked relative changes in the alternative splicing of tau transcripts in adult zebrafish brains. Our results revealed that exposure to acute levels of actual hypoxia or chemical mimicry of hypoxia shifts the production of the predominantly expressed 3R transcript isoform of *maptb* towards formation of the 4R isoform, thus altering the 3R to 4R ratio. The precise regulation of the ratio of expression of 3R relative to 4R MAPT isoforms in human brain has been proposed to be critical for maintaining normal brain function [31]. The disruption of this balance has been found to be correlated with tauopathies [8, 32]. We also observed a significant increase in expression of the 6R transcript isoform of zebrafish *mapta* relative to the *mapta* 4R transcript*.* As far as the behavior in alternative splicing of exons coding for tubulin-binding domain sequences is concerned, our data are in agreement with those of Conrad *et al*. [12] and Ichihara *et al*. showing that, in AD brains, the expression level of exon 10 is altered [33].

Imbalance of the 4R-3R tau isoform ratio has been observed in tauopathies such as FTDP-17 [8], PSP [10], and PiD [34]. An altered 4R-3R tau isoform ratio has also been reported in the spinal cord after sciatic nerve axotomy [35]. Suh *et al.* [28] reported that cerebral ischemia changes the ratio of 4R-3R tau mRNAs and protein levels as well as causing tau hyperphosphorylation. Changes in tau isoform ratio and phosphorylation status can cause defects in the central nervous system by affecting microtubule dynamics and axonal transport resulting in neuronal loss [4]. Therefore, it is conceivable that an alteration of tau isoform ratio and increased tau hyperphosphorylation after brain ischemic insult may contribute to the prevalence of AD in stroke patients [36, 37].

Exon 10 of the human *MAPT* gene, is flanked by a large intron 9 (13.6 kb) and intron 10 (3.8 kb), and has a stem-loop structure which spans the 5′ splice sites, which can sequester the 5′ splice site and leads to the use of alternative 5′ splice sites [38]. Thus exon 10 can be included or skipped to produce tau proteins with or without exon 10, depending on the action of *trans*-acting or *cis-*elements located in exon 10. Hutton M, 1998 [8] The pre-mRNA splicing factor Tra2b was shown to promote *MAPT* exon 10 splicing [39]. Levels of Tra2b protein were found to be reduced in AD brains [12]. Decreased levels of this splicing factor were also observed by Suh *et al*. [28] in cortical neurons and in mouse cerebral cortex following hypoxic-ischemic injury. Thus, decreased Tra2b expression under hypoxia may contribute to a shift in 4R-3R tau isoform ratio by increasing incorporation of exon 10 into mature *MAPT* mRNA. Consistent with this we detected putative Tra2b-binding sites in exon 8 of *mapta* and exon 9 of *maptb.* We also saw decreased expression of *tra2b* mRNA under hypoxic conditions.

High molecular weight (HMW) tau isoforms “big tau” have been detected in the neurons of the adult rat peripheral nervous system (PNS), optic nerve, spinal cord, several neuronal cell lines including PC12 and neuroblastoma N115 [24] and non-neuronal tissues [25, 26]. “Big tau” appears to be the only tau isoform expressed in adult dorsal root ganglia (DRG) [24, 40]. “Big tau” is encoded by an 8 kb mRNA containing an additional exon 4a that is not present in any other tau isoforms. “Big tau” expression is developmentally regulated. It is expressed late in fetal life and its expression increases postnatally [24]. Its presence has been correlated with increased neurite stability in adult DRG [40]. Several studies have investigated “big tau” expression in non-neuronal tissues in AD patients but did not observe any significant changes [25, 26]. Chen *et al.* [23] described an alternative splicing event involving *maptb* exon 3, which appears to be equivalent to human *MAPT* exon 4a. In our experiments we observed that hypoxia significantly increases the level of *maptb* transcripts from which exon 3 sequence is excluded but does not appear to change levels of the “big tau” form of *maptb* transcripts. However, we cannot exclude the possibility that this apparent increase in *maptb* expression with decreased exon 3 inclusion may be due to increased expression of the shorter transcript isoform in cells that do not express big tau, rather than a change in the ratio of splicing to form shorter transcript relative to “big tau” transcript within cells expressing both transcripts.

## Conclusion

Overall, our findings show that exposure of zebrafish brains to actual hypoxia or chemical mimicry of hypoxia can produce changes in the expression ratio of different tau isoforms. These changes are similar to those observed in a number of neurodegenerative diseases and thus support the use of zebrafish as a model for providing further insight into the mechanisms underlying these disease processes.

## Methods

### Ethics

This work was conducted under the auspices of The Animal Ethics Committee of The University of Adelaide and in accordance with EC Directive 86/609/EEC for animal experiments and the Uniform Requirements for Manuscripts Submitted to Biomedical Journals.

### Zebrafish husbandry and experimental procedures

*Danio rerio* were bred and maintained at 28°C on a 14 h light/10 h dark cycle [41]. Adult zebrafish (AB strain) at approximately 1 year of age were used for all experiments (n = 12). Fish for analysis were not selected on the basis of sex. For chemical mimicry of hypoxia adult explant brain tissue was exposed to 100 μM of sodium azide (NaN_3_, Sigma-Aldrich CHEMIE Gmbh, Steinheim, Germany) in DMEM medium for 3 hours. Untreated adult zebrafish brain explants that were dissected from zebrafish in the same way as for the treated adult zebrafish brains were used as *in vitro* controls. In the experiments conducted under low oxygen conditions, oxygen was depleted by bubbling nitrogen gas through the medium. Oxygen concentrations were then measured using a dissolved oxygen meter (DO 6+, EUTECH instruments, Singapore). The dissolved oxygen level in the actual hypoxia group was measured to be 1.15 ± 0.6 mg/l; whereas the normal ambient oxygen level was 6.6 ± 0.45 mg/l [27, 42]. Zebrafish were exposed to actual hypoxia for 3 hours. Briefly, after each hypoxia trial, the animals were euthanized by hypothermic shock and then decapitated to remove the brain. Total RNA was extracted from samples mentioned above using the QIAGEN RNeasy mini kit (QIAGEN, GmbH, Hilden, Germany) and stored at −80°C for further analysis. RNA concentration was determined with a NanoVue™ UV–vis spectrophotometer (GE Healthcare Life Sciences, Fairfield, USA). To insure quality of RNA, RNA samples were electrophoresed on 1% TBE agarose gels. 700 ng of total RNA were used to synthesize 25 μL of first-strand cDNA by reverse transcription (SuperScript® ΙΙΙ First-Strand DNA synthesis kit; Invitrogen, Camarillo, USA).

### Quantitative real-time PCR for detection

The relative standard curve method for quantification was used to determine the expression of experimental samples compared to a basis sample. For experimental samples, target quantity was determined from the standard curve and then compared to the basis sample to determine fold changes in expression. Gene-specific primers were designed for amplification of target cDNA and the cDNA from the ubiquitously expressed control gene e*ef1a1a*. The reaction mixture consisted of 50 ng/*μ*l of cDNA, 18 *μ*M of forward and reverse primers and *Power* SYBR green master mix PCR solution (Applied Biosystems, Warrington, UK).

To generate the standard curve cDNA was serially diluted (100 ng, 50 ng, 25 ng, 12.5 ng). Each sample and standard curve reaction was performed in triplicate for the control gene and experimental genes. Amplification conditions were 2 min at 50°C followed by 10 min at 95°C and then 40–45 cycles of 15 s at 95°C and 1 min at 60°C. Amplification was performed on an ABI 7000 Sequence Detection System (Applied Biosystems) using 96 well plates. Cycle thresholds obtained from each triplicate were averaged and normalized against the expression of *eef1a1l1*, which has previously been demonstrated to show unchanged levels of expression under hypoxia in embryos at 6, 12, 48 and 72 hpf and in adult gills [43]. Each experimental sample was then compared to the basis sample to determine the fold change of expression. The primers used for quantitative real-time PCR analysis of relative zebrafish *mapta/b* mRNA levels are shown in Table 1. To reduce possible interference from unspliced RNA and/or contaminating genomic DNA primers were designed to bind in cDNA over exon-exon boundaries. All qPCRs were performed according to MIQE guidelines [44].

**Table 1:** **Gene specific primers used for qPCR**

Gene/transcript isoform	Accession number	Sequence	Amplicon size
***eef1a1l1 (F)***	**NM_131263.1**	5′-CTGGAGGCCAGCTCAAACAT-3′	**87 bp**
***eef1a1l1 (R)***		5′-ATCAAGAAGAGTAGTACCGCTAGC-3′	
***tra2b (F)***	**NM_201197**	5′-GCAGACGACATATTGGTGACC-3′	**155 bp**
***tra2b (R)***		5′-TGACTGCTGGTCGTACACAATG-3′	
***maptb 4R (F)***	**XM_005171601**	5′-AAGATCGGCTCCACTGAGAACC-3′	**194 bp**
***maptb 4R (R)***		5′-GATCCAACCTTTGACTGGGCTT-3′	
***maptb 3R (F)***	**XM_005171601**	5′-GGGAAGGGGTGGAAATGTC-3′	**140 bp**
***maptb 3R (R)***		5′-GATCCAACCTTTGACTGGGCTT-3′	
***mapta 6R (F)***	**XM_001340530**	5′-TCGTCACAAACCAGGTGGAG-3′	**152 bp**
***mapta 6R (R)***		5′-GCTCACGGAACGTCAGTTTG-3′	
***mapta 4R (F)***	**XM_001340530**	5′-CGGAGGTGGAAAATTGAGTCAC-3′	**100 bp**
***mapta 4R (F)***		5′-CTCCTCCAGGGACACAATTTCT-3′	
***maptb −3 (F)***	**XM_005171601**	5′-GAAGCCAAGGCTGGAGCA-3′	**120 bp**
***maptb −3 (R)***		5′-CTGGGGATGCCTGTGACTGA-3′	
***maptb +3 (F)***	**XM_005171601**	5′-CCGGCAACAACATAGCATCTG-3′	**140 bp**
***maptb +3 (R)***		5′-CACCGGGAGTGAATGTGGC-3′	
***mapta Ex.6 (F)***	**XM_001340530**	5′-CCTAAATCTCCTGCCAGCAAG-3′	**117 bp**
***mapta Ex.6 (R)***		5′-TGTGGGCGAACGGTTCTT-3′	
***maptb Ex.6 (F)***	**XM_005171601**	5′-CAAATCACCTGGCTCGCTG-3′	**114 bp**
***maptb Ex.6(R)***		5′-GGTTGGTGTTTGAGGTTCTCAGTG-3′	

### Statistical analysis of data

Means and standard deviations were calculated for all variables using conventional methods. Two-way ANOVA was used to evaluate significant differences between normoxia and samples from actual hypoxia or chemical mimicry of hypoxia. *p-*Values are shown in the figure legends, a criterion alpha level of *P* < 0.05 was used for all statistical comparisons. All qPCR assays were done in three biological replicates with three qPCRs per biological replicate). All the data were analysed using GraphPad Prism version 6.0 (GraphPad Prism, La Jolla, CA).
